# Spectrum of Surgical Presentation of Eosinophilic Enteritis

**DOI:** 10.1155/2015/691904

**Published:** 2015-04-16

**Authors:** Spoorthy Sudhakar Shetty, Charan Kishor Shetty

**Affiliations:** ^1^Department of Surgery, Faculty of Medicine and Health Sciences, UCSI University, Bukit Kor, PT 11065, Mukim Rusila, 21600 Marang, Terengganu, Malaysia; ^2^Department of Forensic Medicine, Faculty of Medicine, Universiti Sultan Zainal Abidin (Unisza), Kampus Kota, Jalan Sultan Mahmud, 20400 Kuala Terengganu, Terengganu, Malaysia

## Abstract

Eosinophilic enteritis is a rare disorder presenting mostly with diarrhea, malabsorption, abdominal pain, weight loss, and hypersensitivity. Surgical manifestation of eosinophilic gastrointestinal disorders depends on the site and extent of involvement. In our case series of four patients two of them had ileocaecal masses with recurrent subacute intestinal obstruction with past history of intake of antitubercular drugs for 9 months. On histopathological examination both of them proved to have eosinophilic enterocolitis. Thus it is a clinical dilemma to differentiate between these two conditions. The other two patients presented as acute abdomen with perforation and intussusception. All four patients were treated surgically. Postoperatively they recovered well with no symptoms on one year follow-up. In Indian setup tuberculosis being rampant there may be under reporting or wrongly diagnosed cases of eosinophilic enteritis. Thus a strong clinical suspicion and awareness of this clinical entity are essential among surgical community.

## 1. Introduction

Eosinophilic enteritis is a rare disorder presenting mostly with diarrhea, malabsorption, abdominal pain, weight loss, and hypersensitivity. Surgical manifestation of eosinophilic gastrointestinal disorders depends on the site and extent of involvement. Accordingly they can be divided as eosinophilic esophagitis, eosinophilic enteritis, eosinophilic gastritis, and eosinophilic colitis. Eosinophilic gastrointestinal disorders can be primary or secondary to helminthic, fungi, hypereosinophilic syndrome (HES), systemic disease (e.g., connective tissue disease, vasculitis, celiac disease, and inflammatory bowel disease), and drugs (e.g., naproxen, clozapine, rifampicin, and gold) [1]. Presentation tends to be dependent on which intestinal layer is most affected by the eosinophilic infiltration. Mucosa predominant disorder is associated with mucosal injury and presents with malabsorption, diarrhoea, and protein-losing enteropathy. Transmural disease presents with colonic wall thickening and features of intestinal obstruction. Eosinophilic predominant ascites is a manifestation of serosal involvement. Eosinophilic colitis can present acutely with abdominal symptoms such as caecal volvulus causing intestinal obstruction, intussusception, and perforation [2]. Treatment of eosinophilic enterocolitis is antihistamines, mast cell stabilizers, glucocorticosteroids, and immunosuppressive agents along with surgery for acute surgical emergencies.

In Indian scenario where abdominal tuberculosis is widely prevalent, eosinophilic enterocolitis is under reported. Eosinophilic colitis mimics abdominal tuberculosis with identical symptoms of fever, weight loss, abdominal pain, recurrent subacute intestinal obstruction, and ascites. CT scan also has a similar picture of pulled up caecum, strictures, and ascites especially in transmural type of enterocolitis.

## 2. Case Presentation

**Figure 1:**
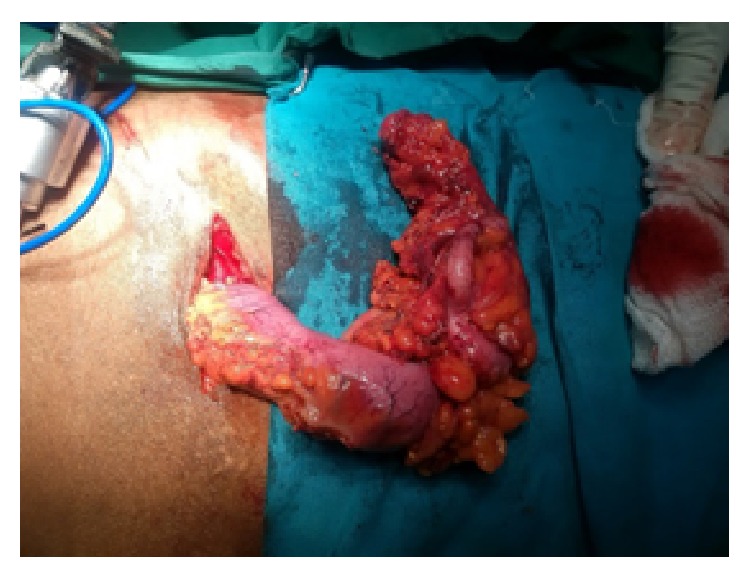
Ileocaecal mass specimen after laparoscopic hemi colectomy (case 1).

**Figure 2:**
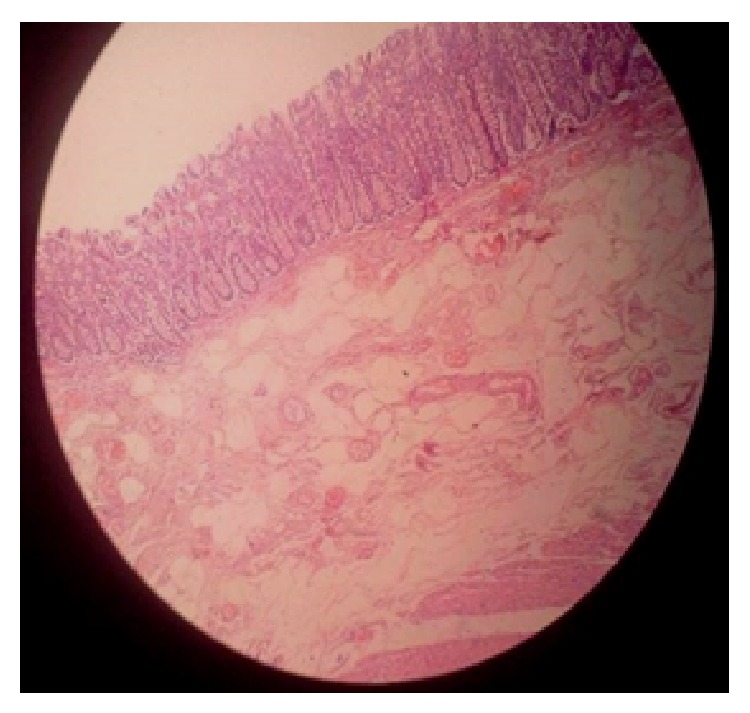
Infiltration of eosinophils is seen in all layers of intestine with large number of intraepithelial eosinophils and eosinophilic micro abscess.

*Case 1*. A 57-year-old male with features of recurrent subacute intestinal obstruction from past one year was evaluated with ultrasonography which showed multiple dilated bowel loops with sluggish peristalsis. We suspected tuberculosis of abdomen as patient gave history of intake of antitubercular drugs for 9 months 1 year back. Patient contrast enhanced CT scan showed thickening in the ileocaecal area with mesenteric lymphadenopathy and dilated bowel loops. Patient had an elevated ESR and lymphocytosis. Patient had diagnostic laparoscopy which showed a pulled up and thickened shrunken caecum along with an ileal stricture. He underwent a right laparoscopic hemicolectomy procedure (Figures 1 and 2). Postoperatively patient recovered well. On histopathological examination of specimen eosinophilic infiltration was seen in all layers of intestine. Patient's absolute eosinophilic count was 550/cumm.

**Figure 3:**
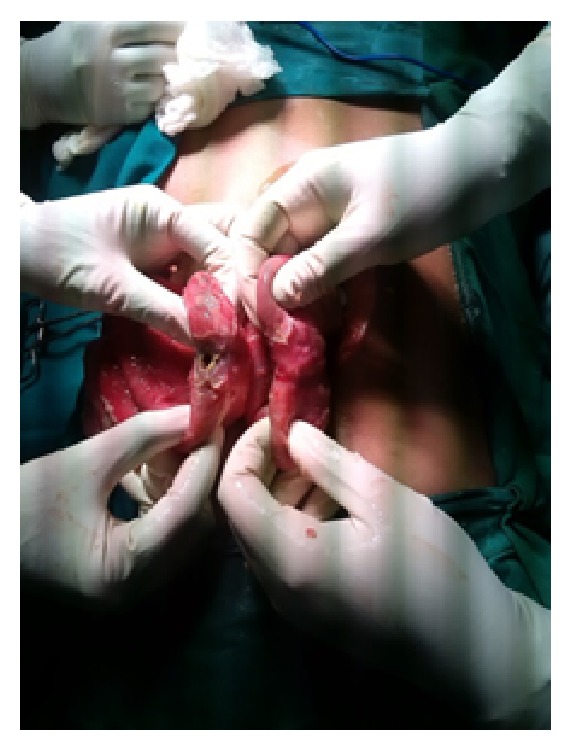
Ileal perforation with stricture (Case 2).

*Case 2*. A 32-year-old male patient came to emergency surgical department with acute abdominal pain. On examination patient had tachycardia with blood pressure of 100/60 mm Hg with high colored urine. Abdominal examination revealed generalized tenderness, guarding, and rigidity. Patients abdominal X-ray erect showed air under diaphragm. Patient underwent an exploratory laparotomy in view of features suggestive of perforative peritonitis. Patient had perforation of ileum with two distal ileal stricture and fibrosed caecum. Ileotransverse bypass was done with closure of ileal perforation after taking biopsy (Figure 3). Postoperatively after histopathological examination patient had eosinophilic enteritis. Patient was given a course of steroid and antihelminthic drugs. Postoperative course was uneventful. We gave steroid in the second patient because we had not done any resective procedure in this patient. We only took multiple biopsies from perforated area and did ileotransverse bypass procedure and left the affected bowel as patient presented in septic shock.

**Figure 4:**
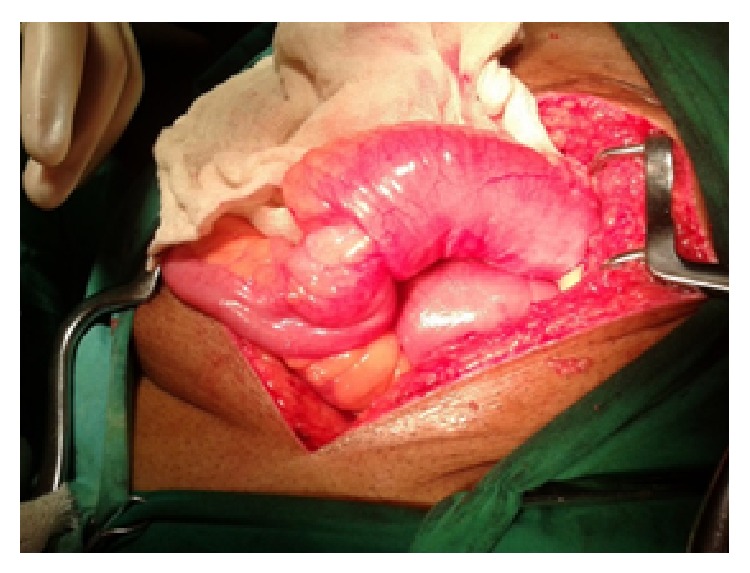
Ileoileal intussusception in a case of eosinophilic enteritis (Case 3).

*Case 3*. A 24-year-old male came with history of constipation, recurrent vomiting, and abdominal distention since 1 day. On evaluation patient had tachycardia with low blood pressure. Patient's abdominal X-ray showed multiple air fluid level and CT scan showed intestinal obstruction with target sign suggestive of intussusception. Exploratory laparotomy was done which showed dilated bowel loops with ileoileal intussusception at the level of distal ileum (Figure 4). Resection anastomosis was performed. Specimen analysis showed eosinophilic enteritis. Patient had elevated absolute eosinophilic count. On one year follow-up patient was symptom-free.

**Figure 5:**
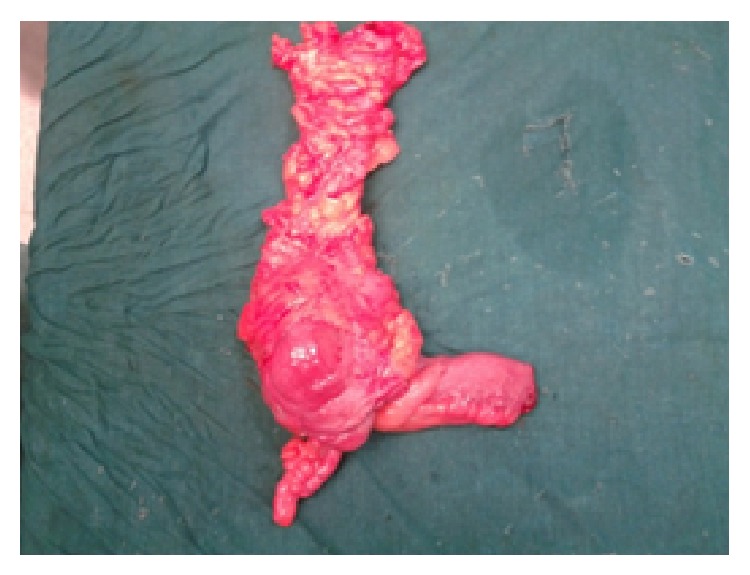
Resected specimen of ileum and caecum after right hemicolectomy (Case 4).

**Figure 6:**
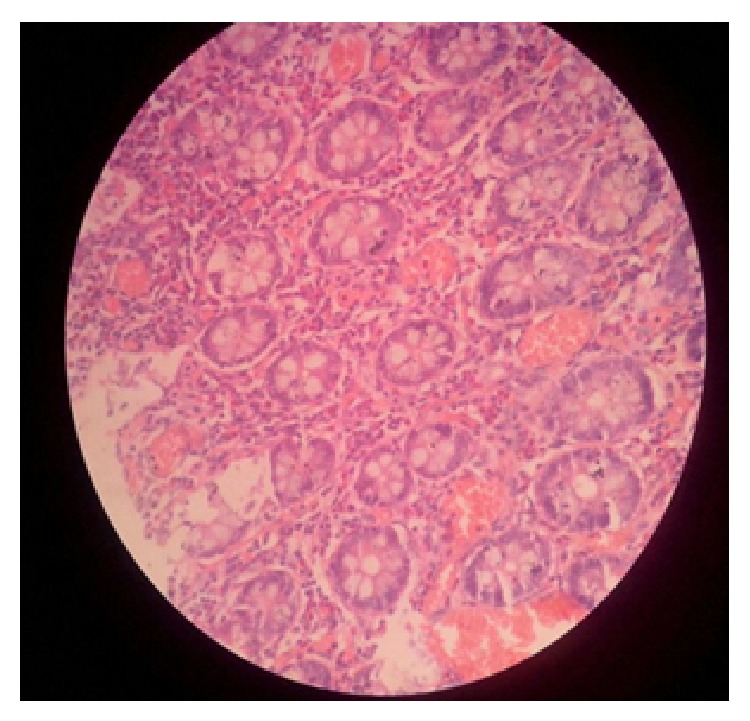
Hematoxylin and eosin stain showing eosinophilic infiltrations (more than 100 cells/HPF) in all layers of intestine (Case 4).

*Case 4*. A 6-year-old male with history of recurrent subacute intestinal obstruction since 3 months was evaluated with ultrasonography which showed multiple dilated bowel loops with sluggish peristalsis. Patients contrast enhanced CT scan showed thickening in the ileocaecal area with dilated bowel loops. Patient had an elevated ESR and lymphocytosis and also gave history of tuberculosis with intake of antitubercular drugs 35 years back for a one year period. Patient had normal eosinophil count. Exploratory laparotomy and right hemicolectomy was done in view of thickened pulled up illeocaecal junction and dilated proximal loops. Postoperatively patient recovered well. On histopathological examination of specimen eosinophilic infiltration was seen in all layers of intestine (Figures 5 and 6). Detailed clinical history, laboratory, and radiological investigations is summarized in (Tables 1 and 2).

## 3. Discussion

Eosinophilic gastrointestinal disease was first described by Kaijser in 1937 [3]. In India, Venkataraman et al. have reported seven cases of EGE over a ten-year period [4]. Diagnosis is one of exclusion and the criteria put forward for the diagnosis are the presence of gastrointestinal (GIT) symptoms, infiltration of the GIT by eosinophils in one or more areas, absence of parasitic infestation, and exclusion of eosinophilic involvement in organs other than the GIT [5]. The diagnosis of EE is made from the presence of gastrointestinal symptoms, peripheral eosinophilia, endoscopic and histological findings, and eosinophilic ascites, with no well-defined causes of eosinophilia on thorough evaluation. In the present case series only two patients had raised peripheral eosinophil count but all had eosinophilic infiltration of >100 cells/HPF on histopathological examination. Hence in a suspected case of EE a colonoscopic biopsy at multiple sites showing eosinophilic infiltration can be one of the diagnostic tools in confirming the disease.

In our case series of four patients two of them had ileocaecal mass with recurrent subacute intestinal obstruction with past history of intake of antitubercular drugs for 9 months. On histopathological examination both of them proved to have eosinophilic enterocolitis. Thus it is a clinical dilemma to differentiate between these two conditions. The other two patients presented as acute abdomen with perforation and intussusception. All four patients were treated surgically. Postoperatively they recovered well with no symptoms on one year follow-up.

Various case reports have been reported where eosinophilic enteritis mimics tuberculosis and ulcerative colitis. In the present case series also out of four patients two of them had a history of abdominal tuberculosis in the past and treatment for the same was taken. Lange et al. proposed rifampicin as a cause of eosinophilic colitis as its side effect [6]. In the present case series it was an enigma to distinguish whether the patients were wrongly diagnosed cases of tuberculosis or were they actual case of eosinophilic enteritis or patient had eosinophilic enteritis as a side effect secondary to the consumption of rifampicin. Both of these patients did not have a proven biopsy suggestive of tuberculosis before starting treatment. According to history they were on anti-tubercular drugs only based on clinical suspicion and CT picture. Drugs reported to cause colonic eosinophilia include nonsteroidal anti-inflammatories, tacrolimus, carbamazepine, rifampicin, sulphasalazine, and naproxen. None of the patients in the case series had any h/o allergic rhinitis or atopy or food allergy. Thus it is essential to conduct colonoscopic biopsy to differentiate between eosinophilic enterocolitis and tuberculosis. Biopsy has to be taken at multiple sites as the disease has tendency to affect as skip lesions.

Eosinophilic enterocolitis also presents as a mass lesion which can be confused with neoplasm. In the third case patient had a lump on CT scan with target lesion which was suspected as malignancy causing a leading point for intussusception but was surprisingly proved as EE. Thus eosinophilic gastrointestinal disorders even though rare have to be kept as differential diagnosis as it is a benign and an easily treatable entity.

Supportive treatment with pharmacotherapy, mainly oral glucocorticosteroids, is indicated for those with obstructive symptoms. Patients with mucosal layer involvement may benefit from anti-inflammatory medications (e.g., oral glucocorticoids) and/or diet elimination therapy, particularly if they report a history of food intolerance or allergy. Drugs, such as montelukast, ketotifen, and mycophenolate mofetil, and alternative Chinese medicines have been advocated but are generally not successful. Recurrence is common even after surgical resection.

## 4. Conclusion

Eosinophilic enteritis even though a rare disorder has a varied spectrum of presentation which is easily misinterpreted as neoplasm, tuberculosis, and inflammatory bowel disease. Since it can be easily treated medically and surgically it has to be diagnosed with certainty. In developing countries, as incidence of Tuberculosis is high it is common to wrongly diagnose case of eosinophilic enteritis as tuberculosis. Thus a strong clinical suspicion and awareness of this clinical entity are essential among surgical fraternity.

## Figures and Tables

**Table 1:** Clinical manifestation, ultrasound findings, and clinical diagnosis.

Number	Age/sex	Symptoms/drug history	Radiological finding	Clinical diagnosis	Laboratory findings
1	57/M	Recurrent constipation Vomiting and distention on and off 1 year Antitubercular drugs for 9 months [CAT 1]	CECT: thickening in ileocaecal area with mesenteric lymphadenopathy with dilated loops	Recurrent intestinal obstruction	ESR: 108 AEC: 550/Cumm Chest X ray: Signs of fibrosis healed tuberculosis Stool examination: NAD Montoux test: negative

2	32/M	Acute abdomen Guarding Rigidity Tachycardia Hypotension No h/o of chronic medication	X-ray erect chest/abdomen showed air under diaphragm	Acute perforative peritonitis	ESR: 100 AEC: 320 cells/cum Chest X-ray: NAD Stool examination: NAD Montoux test: negative

3	24/M	Obstipation, vomiting, and abdominal distention since 1 day No h/o chronic medication	X-ray abdomen: multiple air fluid level CECT abdomen: target sign suggestive of intussusception with obstruction	Acute intestinal obstruction due to ileo ileal intussusception	ESR: 25 AEC: 600 cells/cumm Chest X-ray: NAD Stool examination: NAD Montoux test: negative

4	62/M	Constipation, abdominal distention, and vomiting on and off since past 3 months h/o intake of amlodipine, aspirin, and atorvastatin for 20 years Antitubercular treatment 35 years back	USG: Multiple dilated loops with sluggish peristalsis CECT: thickening in ileo caecal area with proximal dilated loops	Acute intestinal obstruction	ESR: 90 AEC: 70 cells/cumm Chest X-ray: NAD Stool examination: NAD Montoux test: negative

AEC: absolute eosinophil count: normal range: 40–400 cells/cumm.

NAD: no abnormality detected.

ESR: 0–20 mm/hr in male [normal range].

**Table 2:** Management of individual patient.

Number	Medical line of management	Surgical management
1	Resuscitation, IV antibiotics, albendazole, Anti-tubercular treatment, analgesic, pantoprazole, pyridoxine	Laparoscopic right hemicolectomy

2	Resuscitation, IV antibiotics, albendazole, analgesic, and prednisolone oral Pantoprazole	Closure of ileal perforation with biopsy ileo transverse bypass due to multiple ileal stricture and fibrosed caecum

3	Resuscitation, IV antibiotics, albendazole, analgesic, pantoprazole, and tranexamic acid	Resection and anastomosis of intussuscepted ileal segment

4	Resuscitation, IV antibiotics, albendazole, analgesic, pantoprazole, amlodipine, and SC low molecular weight heparin	Exploratory laparotomy with right hemi colectomy
